# Vale Colin Ward—A Leader in Receptor Structural Biology

**DOI:** 10.3389/fendo.2017.00095

**Published:** 2017-05-11

**Authors:** Michael C. Lawrence, Peter M. Colman

**Affiliations:** ^1^The Walter and Eliza Hall Institute of Medical Research, Parkville, VIC, Australia; ^2^Department of Medical Biology, University of Melbourne, Parkville, VIC, Australia

**Keywords:** Colin Ward, insulin receptor, epidermal growth factor receptor, ErbB receptors, hemagglutinin, type 1 insulin-like growth factor receptor

It is with sadness that we report that Colin Ward (Figure 1), our colleague and a world leader in the structural biology of receptor tyrosine kinases, passed away on March 24, 2017.

**Figure 1:**
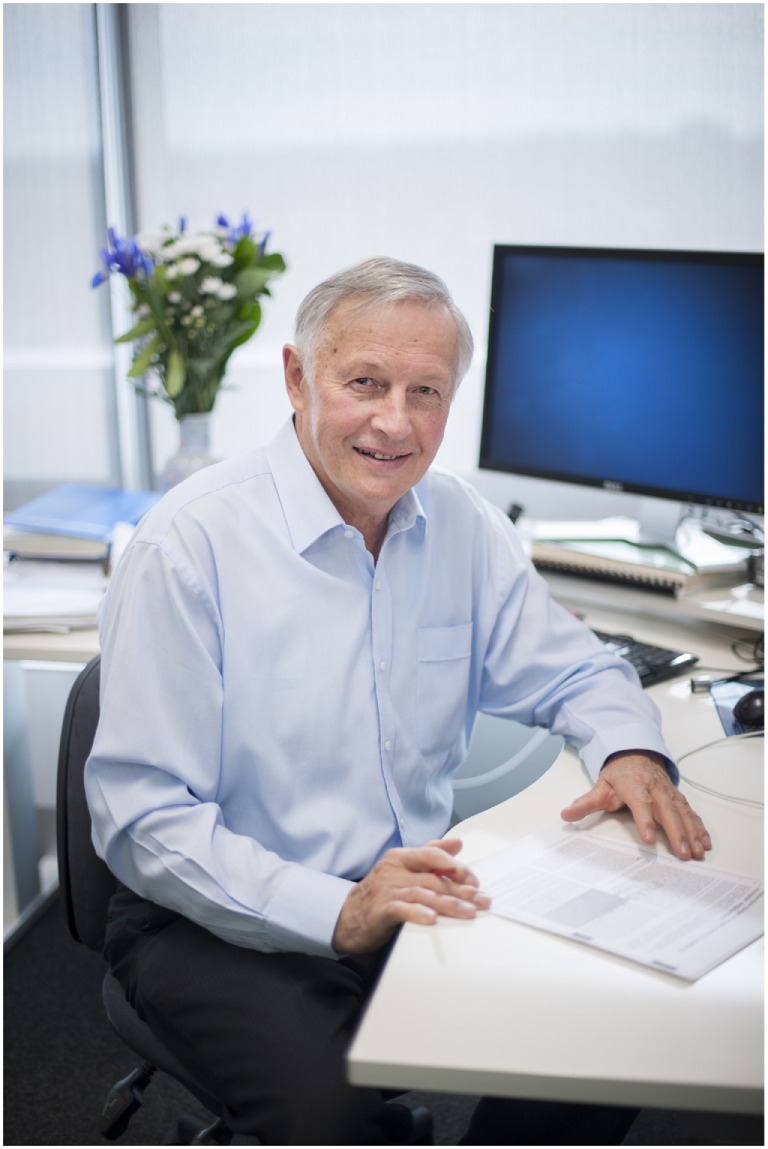
**Dr. Colin Ward, at the Walter and Eliza Hall Institute of Medical Research in 2014**.

Colin was born in 1943 in the rural town of Cootamundra in NSW, Australia. In 1964, he obtained a Bachelor of Science degree with first-class honors at the School of Wool Technology within the University of New South Wales and was also awarded the prestigious University Medal. Although his initial plans were to return to the family farm after graduating, the absence of employment opportunities in the area led to him re-enrolling at the University, obtaining a Ph.D. in 1967. His research was conducted jointly between the School of Wool Technology and the Department of Biochemistry and concerned the biochemistry of the carbohydrate metabolic pathways of parasitic nematodes. His interest in this area continued during a two-year postdoctoral period within the Department of Zoology at the University of Massachusetts in Amherst.

Upon his return from the USA in 1970 to Australia, he joined the Division of Protein Chemistry as a Research Scientist within the Commonwealth Scientific and Industrial Research Organisation (CSIRO) in Parkville, Melbourne. His first project was aimed at understanding how keratin protein of wool was enzymatically digested by insect moth larvae. At that time, Australia was very dependent on the export of wool and a considerable part of the effort within the Division was devoted to understanding the molecular structure of keratins. Colin characterized over 29 enzymes within the larval gut, but by then, it was time to move on to more ambitious goals.

In 1972, he began research into the influenza virus, culturing the virus over the following 9 years in over 150,000 chick embryos. Together with his colleagues, he obtained sequences of the hemagglutinin protein from two human Hong Kong strains and one bird virus, the latter later believed to be the precursor of the 1968 Hong Kong pandemic. These data informed much of the understanding of antigenic variation within the virus and ultimately its structural biology. In 1980, Colin predicted that the hemagglutinin contained a coil-coiled element, an observation undoubtedly linked to his knowledge of keratin, also a coiled-coil protein (1). The key role of this coiled coil in viral fusion was finally laid bare in 1994, with its link to Colin’s predictions being recorded in the associated *Nature: News and Views* (2). Again together with colleagues, he determined the sequence of the viral neuraminidase, its disulfide bond structure and its glycosylation pattern. This work was integral to the subsequent determination, by Peter Colman and Jose Varghese, of the protein’s three-dimensional structure, in turn leading to the development of the anti-influenza drug, zanamavir.

His research then moved into other areas relevant to agriculture, including establishment of the definitive taxonomy of the Potyviruses (3), elucidation of the serotypes of *Dichelobacter nodosus* (the causative organism of ovine footrot), and development of a vaccine against infectious bursal disease virus, a major pathogen in poultry industries.

Colin finally turned his interest to the structural biology of the insulin receptor, a passion that was to persist for the remainder of career, with part of his interest stemming from the fact that a family member suffered from juvenile diabetes. The insulin receptor, like its partner proteins—the type 1 insulin-like growth factor receptor (IGF-1R) and the insulin receptor-related receptor—are disulfide-linked, homodimeric (αβ)_2_ proteins. These proteins contain a very high number of intra-molecular disulfides and are extensively glycosylated, themes reminiscent of his earlier research. Understanding their structural biology has widely been recognized as particularly challenging. Colin set about assembling a team within CSIRO that was built up of many of his erstwhile colleagues and together they elucidated the nature of the disulfide bonds in the insulin receptor. They also established in the 1990s a large-scale mammalian cell culture facility to produce the protein and later in the early 2000s the first robotic protein crystallization facility in Australia. Particular attention was paid to obtaining and purifying large quantities of receptor constructs to isoelectric purity, a feature deemed to be the key to obtaining high-quality, diffracting crystals. The advent of large-scale mammalian culture led to other notable structural biology successes along the way, including the first structures of (a) the first three domains of IGF-1R (4), (b) the ectodomain of the epidermal growth factor receptor in complex with transforming growth factor α (5), (c) the ectodomain of ErbB2 (6), and (d) the first three domains of the insulin receptor (7). Shortly before his retirement in 2006, his group finally published the structure of the intact insulin receptor ectodomain (8, 9), immediately acknowledged as being of landmark importance.

Co-incidentally, in 2006, CSIRO withdrew support from insulin receptor research. Recognizing that the goal of understanding how insulin engages its receptor remained unfulfilled, Colin sought a new home for his research, namely, at the Walter and Eliza Hall Institute of Medical Research (WEHI), also in Parkville. Colin’s involvement with the WEHI-based research was now largely “hands-off,” selflessly mentoring his colleague Mike Lawrence to assume the on-going leadership of the project. The move to WEHI enabled them to recruit the involvement of insulin receptor experts outside of Australia (Mike Weiss, Jonathan Whittaker, Don Steiner, Shu Jin Chan, Guy Dodson, Marek Brzozowski, and Jiří Jiráček) to the project and together in 2013 they published the first structure of insulin bound to its primary binding site on the receptor surface (10).

Colin’s research output, evidenced in the authorship of approximately 200 papers, led to many awards. In particular, he was thrice awarded the prestigious CSIRO’s Chairman’s Medal (the only person to have won the award that many times) and also awarded the Lemberg Medal of the Australian Society of Biochemistry and Molecular Biology. He was an elected fellow of the Australian Academy of Science, of the Australian Academy of Technological Sciences and Engineering, and of the Australian Institute of Agricultural Science. His broader interest in Australian science led him to develop an on-line resource, CSIROpedia (11) that enabled the recording of the achievements of CSIRO and its scientists in a publically accessible fashion.

Colin also had many interests beyond research, in particular, rugby, in which he had excelled as a university student. He was devoted to his wife, Lyn, and to his two children and five grandchildren. In retirement, he published a history of his family in Australia. His broad-reaching scientific leadership and his unmitigated enthusiasm, acumen, and friendship will be greatly missed by all who knew him.

## Author Contributions

All authors listed have made substantial, direct, and intellectual contribution to the work and approved it for publication.

## Conflict of Interest Statement

The authors declare that the research was conducted in the absence of any commercial or financial relationships that could be construed as a potential conflict of interest.
